# Philadelphia College of Dentistry

**Published:** 1852-01

**Authors:** 


*Philadelphia College of Dentistry.*—Under a charter, granted by the legislature of Pennsylvania, for a college of Dentistry at Philadelphia, a faculty was elected on the 17th of October, 1851, composed of five professors, namely:

J. D. White, M. D., Professor of Anatomy and Physiology.

Ely Parry, M. D., Professor of Dental Chemistry, Therapeutics and Materia Medica.

Elisha Townsend, D. D. S., Professor of Operative Dentistry and Dental Hygiene.

Robt. Arthur, D. D. S., Professor of Principles and Practice of Dental Surgery.

J. L. Buckingham, Professor of Mechanical Dentistry.

Most of the above named gentlemen are known to the profession as men of ability, and all, we doubt not, are well qualified to discharge the duties respectively assigned to them. The first course of instruction, we understand, will commence next fall, and, under the auspices of so able a faculty, even though it may require years of gratuitous labor, we cannot doubt but that the enterprise will ultimately be crowned with success. It will probably require some twelve or fifteen years, to effect a sufficient change in public opinion to render it necessary, that those to whom the management of the diseases of the mouth is committed, shall have been properly educated for the business, but this change will certainly take place; it has already commenced, and when it shall have become general, the number of dental students who will then avail themselves of collegiate instruction will be sufficient to remunerate, moderately at least, the teachers of three or four schools in the United States. We say this, that those who have recently become interested in furnishing facilities for instruction in dental surgery may not become discouraged, but that they may look rather to the elevation of the standard of professional qualification, than immediate pecuniary reward. We hail the establishment of every new college of dental surgery as an indication of the rapid progress of the change to which we have referred; and most cordially do we tender to the professors composing the faculty of the one in Philadelphia, our best wishes for their success, hoping the day may not be distant, when they will reap a rich reward for the labors in which they are about to engage.

